# Simultaneous treatment of large hiatal hernias during Roux-en-Y gastric bypass: technical considerations and outcome

**DOI:** 10.1007/s13304-024-02017-9

**Published:** 2024-10-02

**Authors:** Lars Kollmann, Annette Thurner, Alexander Dimitri Miras, Florian Seyfried

**Affiliations:** 1 Head of Surgery for Upper Gastrointestinal Tract and Metabolic Surgery, Department of General, Visceral, Transplantation, Vascular and Pediatric Surgery, Center of Operative Medicine (ZOM), University Hospital of Würzburg, Würzburg, Germany; 2 Department of Radiology, University Hospital Wuerzburg, Würzburg, Germany; 3 School of Medicine, Ulster University, Derry, UK

**Keywords:** Upside down stomach, morbid obesity, large hiatal hernia, simultaneous treatment, Roux-en-Y gastric bypass

## Abstract

Roux-en-Y gastric bypass (RYGB) is the preferred surgical option for patients with proven gastroesophageal reflux disease and obesity grade ≥ II (BMI ≥ 35 kg/m^2^). Data on simultaneous treatment of larger hiatal hernias during RYGB are scarce. From 2012 until 2022, data from all consecutive patients undergoing gastric bypass procedures were collected and retrospectively analyzed. The characteristics and surgical outcomes of patients undergoing RYGB alone (RYGBa) versus RYGB with simultaneous treatment of a large hiatal hernia (RYGB-HH) were compared. Out of 573 patients who received RYGB, we identified 12 simultaneously treated for large hiatal hernia. The characteristics of RYGB-HH versus RYGBa patients were higher age (55 vs. 44 years; *p* = 0.004) and lower BMI (39.2 vs. 46.9 kg/m^2^; *p* = 0.001). Duration of surgery in the RYGB-HH group was longer (144 min vs. 98 min; *p* < 0.001), while complications > Clavien–Dindo II were similar compared to the RYGBa group (8.3 vs. 9.4%, *p* = 0.56). Length of stay did not differ among the groups (4 vs. 5.5 days, *p* = 0.051). At a median follow-up of 12 months, there was no clinical recurrence of hiatal hernia in the RYGB-HH group. Simultaneous treatment of large hiatal hernias during Roux-en-Y gastric bypass surgery prolongs operation time but seems feasible and safe in the hands of experienced surgeons.

**Supplementary Information:**

The online version contains supplementary material available at 10.1007/s13304-024-02017-9.

## Introduction

Roux-en-Y gastric bypass (RYGB) is the preferred surgical option for patients with proven gastroesophageal reflux disease and obesity grade ≥ II [1, 2]. Treatment of large hiatal hernias during RYGB in patients with obesity can be challenging, while data on the feasibility and safety are scarce [3].

We aimed to evaluate simultaneous treatment of large hiatal hernias alongside RYGB and to provide information on the setup and technical considerations to perform these surgeries safely.

## Material and methods

All bariatric patients were treated according to international guidelines [4]. Patients with large hiatal hernias (Type IV; > 5 cm length) and BMI > 35 kg/m^2^ were offered simultaneous hiatal hernia repair during RYGB. Esophago-gastro-duodenoscopy was performed prior to bariatric surgery in all patients.

### Radiological diagnostics

Radiological assessment was performed by an experienced radiologist using syngo.via^™^ post-processing software with the auto-contouring algorithm 3D Lesion Segmentation (Siemens Healthcare GmbH, Erlangen, Germany).

### Surgical technique

All surgeries were performed laparoscopically in beach chair position by an experienced upper gastrointestinal and certified bariatric surgeon. Four trocars and a liver retractor (Mediflex^®^) were used.

Roux-en-Y gastric bypass was created in an antecolic and antegastric fashion, with the greater omentum being divided. The length of the Roux and biliopancreatic limb was 120 cm and 80 cm, respectively. The entero-entero-anastomosis (EEA) was created side to side using a linear stapler.

In the case of a large hiatal hernia, a full dissection of the hernia sac was performed until tension-free intra-abdominal positioning of the gastroesophageal junction was accomplished. If a pleural lesion occurred, the capnoperitoneum pressure was reduced, while the positive end-expiratory pressure (PEEP) was increased accordingly to prevent the necessity of a chest tube. During full dissection of the hernia sac, the short gastric vessels were preserved to maintain sufficient blood supply to the fundus. A posterior hiatoplasty was performed, while a 36 Charriere bougie was used for calibration.

A small pouch (30 mL) was created, and a side-to-side gastro-jejunostomy (GJ) using a linear cutting stapler was performed. The defect was closed with running resorbable 3/0 sutures. The mesenteric gaps were closed with running non-resorbable sutures.

### Data assembly and statistical analyses

From January 2012 until December 2022, 573 consecutive RYGB surgeries were performed. Of those, 12 patients received simultaneous treatment for a large hiatal hernia (Hill Type IV; > 5 cm length).

The primary end point was overall complication measured by the Clavien–Dindo-comprehensive complication index [5]. Secondary end points were duration of surgery, reoperation rate, length of stay, and recurrence of hiatal hernia.

Statistical analyses were performed using IBM SPSS Statistics 29 (International Business Machines Corporation, Armonk, NY). Descriptive data were reported as means with standard deviations, unless otherwise stated. Comparisons between the analyzed cohorts were performed using Chi-square, Fisher’s exact, and Mann–Whitney *U* tests or one-way analysis of variance, in accordance with data scale and distribution. The level of statistical significance was 0.05.

## Results

### Patient characteristics

The patients’ characteristics are presented in detail in Table 1.

**Table 1:** Patients’ characteristics and operative outcome

Patients’ characteristics	RYGB (*n* = 561)	Hiatal hernia + GB (*n* = 12)	*p*-value
Sex ratio, no. (M:F)	136:425 (24:76.)	3:9 (25/75)	0.524
Age, mean (SD), y	44 (17–69)	55 (42–69)	0.004
BMI, mean (SD), kg/m^2^	46.9 (34.1–81.0)	39.2 (35.0–46.3)	0.001
Charlson comorbidity score, mean (SD)	1 (0–10)	2 (1–5)	0.02
ASA classification ≥ III	306 (54.5)	6 (50)	0.377
Edmonton obesity scoring system (EOSS) ≥ III	131 (23.4)	2(16.7)	0.270
Type 2 diabetes mellitus	218 (38.9)	4 (33.3)	0.344
Insulin-dependent (IDDM)	48 (8.6)	0 (0)	< 0.001
Obstructive sleep apnea (OSAS)	165 (29.4)	5 (41.7)	0.197
Liver steatosis	82 (14.6)	0 (0)	< 0.001
Chronic kidney failure	18 (3.2)	0 (0)	< 0.001
Perioperative outcome			
Duration of surgery	98 (61–152)	144 (109–242*)	< 0.001
Postoperative complications (CDC > grade II)	53 (9.4)	1 (8.3)	0.555
Reoperation	26 (4.6)	1 (8.3)**	0.323
CCI, mean (mean 95% CI)	3.7 (0–70.6)	0 (0–33.7)	0.287
LOS (mean 95% CI)	5.5 (4–8)	4 (3–13)	0.051

*No*. number; *M* male; *F* female; *SD* standard deviation; *y* years; *BMI* body mass index; *CDC* Clavien–Dindo classification; *CI* confidence interval; *CCI* comprehensive complication index; *LOS* length of stay

*One case of recurrent upside-down stomach with prolonged mediastinal adhesiolysis

**One patient developed an umbilical hernia unrelated to the previous surgery in the 30-day period

### Radiological measurement of hiatal hernia size

**Fig. 1:**
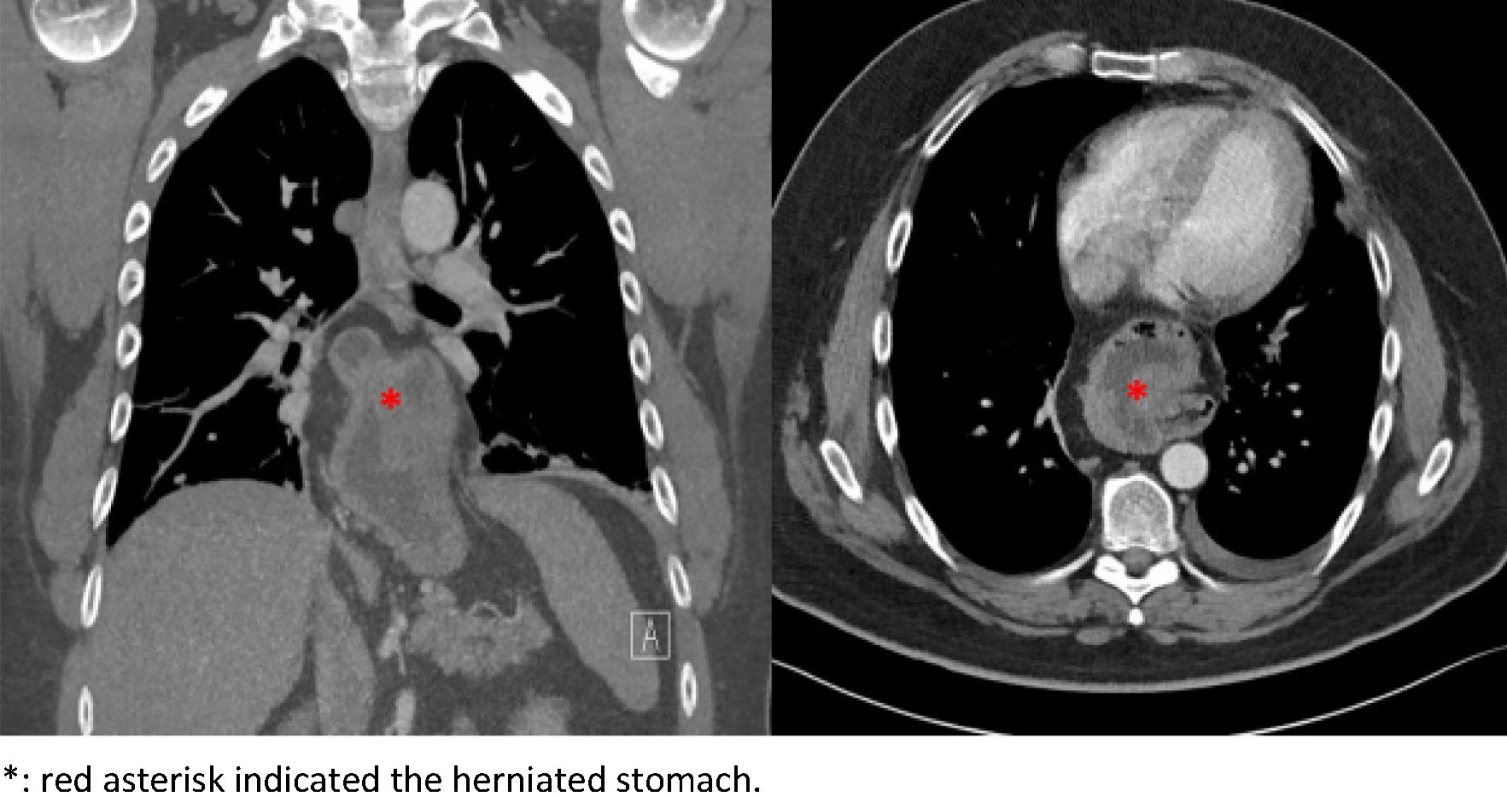
Exemplary CT scan of a patient with upside-down stomach and a BMI of 40 kg/m^2^

The radiological measurements are presented in Table 2. An exemplary CT scan is shown in Fig. 1.

**Table 2:** Radiological characteristics of hiatal hernia cases

Radiological characteristics	Hiatal hernia (*n* = 12)
Diagnostic modality: computed tomography (CT)	10 (83.3)
Hernia type > III	12 (100)
Complete upside-down stomach	3 (25)
Diameter hiatal gap (median, range) in cm	4.5 (3–6)
Volume hernia (in mL) (median, range)	510 (100–1855)
Volume of herniated stomach (in mL; median, range)	334 (98–510)
Volume of intra-abdominal stomach (in mL; median, range)	122 (5–440)
Volume of stomach total (in mL; median, range)	387 (243–846)
% of stomach herniated	63 (24–99)

### Upper gastrointestinal endoscopy

All patients in the RYGB-HH group (*n* = 12) had large hiatal hernias (Hill grade IV, > 5 cm in length), while three (25%) had complete upside-down stomach.

### Operative outcome

The detailed data for the operative outcome are shown in Table 1.

### Follow-up

The median follow-up in the RYGB-HH group was 12 months (range 2–60). The median total body weight loss (TBWL) was 19.3% (11.5–34.4%). No patient reported GERD symptoms or dysphagia. There were no revisional surgeries in the upside-down-stomach group during follow-up.

## Discussion

Our results indicate that simultaneous treatment of large hiatal hernias during Roux-en-Y gastric bypass surgery prolongs operative time but is feasible and safe.

Hiatal repair and fundoplication for upside-down stomach has been shown to contain a recurrence risk of up to 30% in patients with obesity and is, therefore, not recommended in these situations [6, 7]. In contrast, convincing evidence demonstrated the superiority of RYGB for GERD control and recurrence in these situations [8].

Up to now, limited data on simultaneous treatment of large hiatal hernias during RYGB have demonstrated long operative times (234 and 241 min, respectively) but satisfactory outcome and safety [9, 10]. Of note, available studies also include a large amount of grade III hiatal hernias. In our cohort, we only included patients with partial or total upside-down stomach as seen in the CT-volumetry with median 63% of the stomach herniated among the cases.

To achieve successful and safe simultaneous hiatal repair and RYGB, we included a video (Supplement 1) but also would like to highlight the following procedure-specific details:

1. Exploration of the small intestine and the jejunojejunostomy should be performed first. Thereafter, the pressure of the capnoperitoneum can be reduced and matched to the positive end-expiratory pressure in the event of pleural injury to avoid the necessity of thoracic drains.
2. The short gastric vessels should be preserved if possible. While the left gastric vessels are mandatory for sufficient blood supply of the pouch, it might become necessary to partly dissect the short gastric vessels during the reposition of the stomach. In the case of signs of ischemia of the fundus, the resection of the fundus at a securely identified area of vital tissue may be necessary.

## Conclusion

Simultaneous treatment of large hiatal hernias during gastric bypass surgery in patients with obesity is feasible and safe in experienced hands. Our video may be helpful to achieve acceptable operation times and to avoid intraoperative and perioperative complications.

## Supplementary information

Below is the link to the electronic supplementary material.

Supplementary file1 (DOCX 12 KB)

## Abbreviations

RYGB: Roux-en-Y-gastric bypass
RYGB-HH: Roux-en-Y-gastric bypass + hiatal hernia
GERD: Gastroesophageal reflux disease
BMI: Body mass index
MRI: Magnetic resonance imaging
EEA: Entero-entero-anastomosis
PEEP: Positive end-expiratory pressure
IPOM: Intraperitoneal onlay mesh
GJ: Gastro-jejunostomy
CT: Computed tomography
QoL: Quality of life
TBWL: Total body weight loss
