# Supplementary material for: Simultaneous treatment of large hiatal hernias during Roux-en-Y gastric bypass: technical considerations and outcome
Source: Updates Surg. 2024 Oct 2;76(8):2973–6. doi: 10.1007/s13304-024-02017-9 (PMC11628427; doi:10.1007/s13304-024-02017-9)
Supplement: Supplementary file 1 — Supplementary file1 (DOCX 12 KB) [file 13304_2024_2017_MOESM1_ESM.docx]

**Supplements:**

Supplement 1: Video of upside down-stomach reposition with simultaneous RYGB.

Video link: <https://fex.ukw.de/public/download-shares/SioMKtpL5tOOI0118oiTPHyqYCB8VxuL>

Password: Thoraxmagen_1
